# Genetic testing for Lynch syndrome: family communication and motivation

**DOI:** 10.1007/s10689-015-9842-8

**Published:** 2015-10-07

**Authors:** Celine H. M. Leenen, Mariska den Heijer, Conny van der Meer, Ernst J. Kuipers, Monique E. van Leerdam, Anja Wagner

**Affiliations:** Department of Gastroenterology and Hepatology, Erasmus MC, University Medical Center, ‘s-Gravendijkwal 230, 3015 CE Rotterdam, The Netherlands; Department of Clinical Genetics, Erasmus MC, University Medical Center, Rotterdam, The Netherlands; Department of Medical Psychology and Psychotherapy, Erasmus MC, University Medical Center, Rotterdam, The Netherlands; Department of Internal Medicine, Erasmus MC, University Medical Center, Rotterdam, The Netherlands; Department of Gastroenterology and Hepatology, National Cancer Institute, Amsterdam, The Netherlands

**Keywords:** Lynch syndrome, Genetic testing, Family communication, Motivation

## Abstract

Current genetic counselling practice for Lynch syndrome (LS) relies on diagnosed index patients to inform their biological family about LS, referred to as the family-mediated approach. The objective of this study was to evaluate this approach and to identify factors influencing the uptake of genetic testing for LS. In 59 mutation carriers, 70 non carriers and 16 non-tested relatives socio-demographic characteristics, family communication regarding LS, experiences and attitudes towards the family-mediated approach and motivations for genetic testing, were assessed. The majority of all respondents (73 %) were satisfied with the family-mediated approach. Nevertheless, 59 % of the respondents experienced informing a family member and 57 % being informed by a family member as burdensome. Non-tested differed from tested respondents, in that they were younger, less closely related to the index patient and a lower proportion had children. The most important reasons for declining genetic testing were (1) anticipating problems with life insurance and mortgage, (2) being content with life as it is, and (3) not experiencing any physical complaints. In conclusion, the majority of respondents consider the current family-mediated information procedure acceptable, although the provision of information on LS by relatives may be burdensome. Special attention should be paid to communication of LS to more distant relatives.

## Introduction

Lynch syndrome (LS) is a hereditary condition which predisposes to colorectal cancer, endometrial cancer and other cancers [1, 2]. It is caused by inherited germline mutations in mismatch repair (MMR) genes *MLH1, MSH2, MSH6* and *PMS2* or the *EPCAM* gene [3–8]. LS carriers have an increased cumulative lifetime risk for colorectal cancer of 25–70 %, while women with LS carry a lifetime risk to develop endometrial cancer of 13–65 % [9–20]. In addition, LS carriers have an increased risk for cancers of the stomach, ovaries, small bowel, urinary tract, skin and brain [21–24].

Genetic testing for LS is available to all family members of a mutation carrier. Genetic testing can have medical and psychological advantages, irrespective of the outcome in an individual subject. Non-carriers may avoid unnecessary surveillance programs for LS and experience relief from worries about developing cancer both for themselves and their children. For carriers, genetic testing can lead to relief from uncertainty and guide screening recommendations, improving survival through early detection [21, 25]. Despite the potential benefits of genetic testing, a Dutch study on the interest in genetic testing for hereditary colorectal cancer syndromes showed that almost half of the subjects in this cohort of family members at risk did not opt for genetic testing for LS at a median follow-up time after identification of the family specific mutation of 82 months, ranging 10–140 months [26].

In the Netherlands the communication regarding presence of a LS gene mutation within a family occurs by means of the family-mediated approach. When a pathogenic mutation is detected the counselee is asked to inform all at risk relatives. During the counselling process, communication strategies to inform relatives are discussed with the counselee. Furthermore a letter to inform relatives is supplied. This approach implies that family members are responsible to inform their relatives on the diagnosis of LS and the possibility of genetic testing. Currently, little is known about patients’ experiences with and attitudes towards this family-mediated approach [27]. Knowledge on the experiences and challenges with regards to informing family members may help to improve counselling procedures. A previous US study on family communication of LS genetic test results showed that most individuals who undergo genetic testing for LS share their test result with first degree relatives, while more distant relatives are reached less often [28]. Interestingly, a Finnish study on family communication of LS-genetic testing results showed a significant gender difference. Men were less likely to communicate the diagnosis of LS to their relatives, yet disclosed this result significantly more often via a support person such as a spouse [29]. A previous qualitative study in the Netherlands among 30 individuals from LS families showed that motivation to disclose seemed to increase if there were more cancer cases in the family. Disrupted family relations were found to be an important reason for non-disclosure. The way family members communicate about LS may also influence whether or not at-risk family members decide to opt for genetic testing [30, 31]. It would be of clinical interest to gain more insight into the factors influencing the decision whether or not to opt for genetic testing. However, clinical information about the group of non-tested individuals for LS is lacking, since individuals who do not opt for genetic testing often do not apply for genetic counselling.

The aims of this study were to (1) evaluate experiences and attitudes towards a family-mediated approach in an LS cohort, (2) compare tested (mutation carriers and non-carriers) and non-tested individuals on demographic characteristics, anxiety, cancer worry, medical history, family communication, experiences and attitudes towards the family mediated approach, and (3) explore the motivations for uptake or decline of genetic testing for LS.

## Methods

### Subjects and procedure

We conducted a cross-sectional survey among individuals with a personal or family history of LS. The study was performed at the Department of Clinical Genetics of the Erasmus MC University Medical Center. Subjects were recruited from a cohort of 40 LS families with a proven LS mutation. All individuals were 25 years or older, since it is recommended to undergo genetic testing after this age. The tested individuals had received their genetic test result between 1995 and 2009. For each individual a family pedigree was available with detailed medical information.

Two hundred ninety seven tested individuals ≥25 years of age, including index patients, from the above described LS cohort were notified about the start of the research project by an advanced notification letter. Individuals who were interested in participating were asked to respond via a reply card and were subsequently contacted by de study coordinator. The study coordinator informed the individual about the study and asked the individual to participate in this survey. In addition, the study coordinator specifically asked the tested individuals if they knew family members who had refrained from genetic testing for LS. The tested individuals were asked to contact these non-tested family members, in order to obtain consent for being approached for research purposes. A questionnaire was sent to all individuals who consented to participate. Individuals who did not return the questionnaire after two follow-up telephone calls and two additional mailings were considered non-responders.

This study was approved by the Institutional Review Board of The Erasmus MC, and written informed consent was obtained from all respondents.

### Measures

The self-reported questionnaire addressed socio-demographic characteristics including age, gender, marital status, number of children, level of education, employment and medical characteristics.

In addition, respondents were asked whether they, themselves or their relatives had ever been diagnosed with cancer, and to indicate the degree of relatedness to the closest relative affected by cancer. Medical data of tested respondents was cross-checked with their family pedigree at the Department of Clinical Genetics.

Family communication regarding LS was evaluated by a list of questions developed by the authors after a literature search [30, 32–34]. Respondents were asked who informed them about LS, when they were informed, in which way and how the contact was before en after disclosure of the LS diagnosis. Furthermore we asked if it was burdensome to be informed and/or informing relatives on LS using a five-point Likert scale with response options ranging from 1 ‘*very burdensome’* to 5 ‘*not burdensome’*.

Attitude towards the family mediated approach was measured by a self-developed questionnaire with two statements regarding moral duty to disclose LS diagnosis and two questions where respondents was asked if they were satisfied with the current family mediated approach. These two questions had multiple response options including “other”.

Anxiety and depression were measured by the Hospital Anxiety and Depression scale (HADS). Seven items of the HADS reflect anxiety and seven reflect depression. Response options range from 0 to 3 [35]. The sum on each subscale indicates the overall anxiety and depression score (between 0 and 21). A sum score of 11 or more is the threshold for clinical anxiety.

We assessed concerns regarding cancer by means of the cancer worry scale (CWS) [36]. The CWS is a four-item scale that measures worries about the risk of developing cancer and the impact of worries on daily functioning (frequency of thoughts of developing cancer, impact of thoughts about cancer on mood, impact of thoughts about cancer on daily activities, and level of concern for developing cancer). Each item has four possible responses (from 1 *‘not at all’*, to 4 *‘almost all the time/very concerned’*), which are summed to create a CWS between 4 and 16. A higher score indicates more concerns regarding cancer.

Motivation for genetic testing was evaluated using a list of 15 reasons for non-participation, which was adapted from literature [37, 38]. Non-tested respondents were asked to rate to what extent they agreed with these reasons for non-participation in genetic testing on a five-point Likert scale with response options ranging from 1 *‘totally disagree’* to 5 ‘*totally agree’*. An open field was included to add another reason for non-participation.

The questionnaire was pilot tested among ten LS carriers visiting the outpatient clinic.

### Statistical analysis

Categorical variables were used to calculate proportions and interquartile ranges. The association between categorical variables was examined by means of the Chi squared test or Fisher’s exact test. For ordered categorical variables, the Mann–Whitney test was used. Scores from the HADS and cancer worry were treated as continuous variables. For continuous variables the mean and standard deviation was calculated. These variables were tested using the independent sample *T* test. Respondents with missing data were omitted from the respective analyses.

Mutation carriers, non-carriers and non-tested respondents were compared on socio-demographic characteristics, anxiety, cancer worry, medical history, family communication, experiences and attitudes towards the family mediated approach. SPSS 17.0 statistical package was used to analyse data. All *p* values are two-sided and a *p* value of <0.05 was considered significant.

## Results

### Subject characteristics

**Fig. 1:**
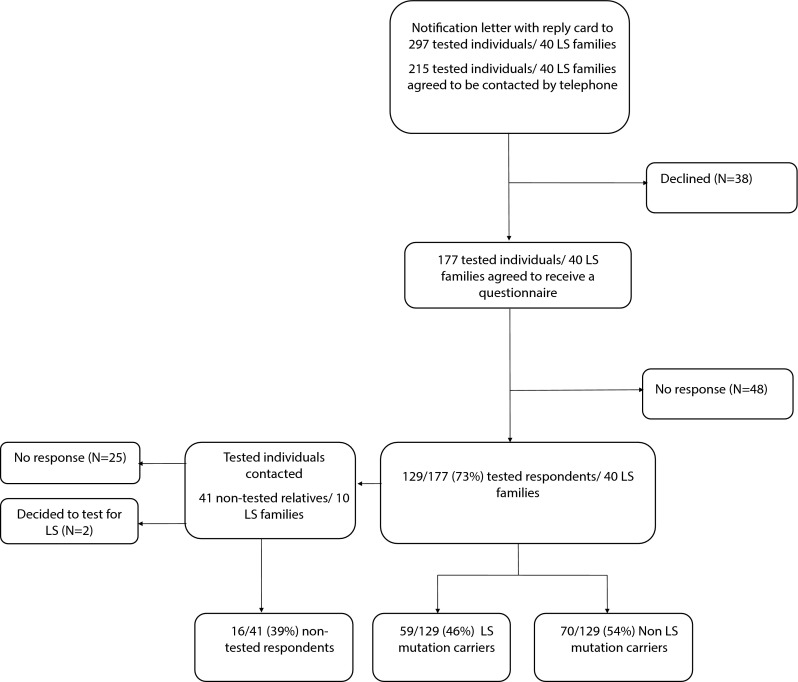
Flowchart of the study procedure

Two hundred ninety seven eligible individuals were approached for enrolment by an advanced notification letter with reply card. Of these, 215 (72 %) agreed to be contacted by phone (Fig. 1). Of the 215 subjects who agreed to be contacted, 177 (60 %) accepted to receive the questionnaire. One-hundred and twenty-nine (43 %) tested individuals from 33 LS families returned the questionnaire. A total of 41 non-tested individuals were contacted via the tested individuals and 18/41 (44 %) non-tested individuals returned the questionnaire. Two non-tested individuals were excluded, since they underwent genetic testing before completing the questionnaire.

There was no difference in age and gender between non-participants, non-responders and responders in the tested and non-tested group (data not shown).

Baseline characteristics of all 145 respondents are shown in Table 1. Of all 129 tested respondents, 59 (46 %) were mutation carriers and 70 (54 %) had no LS mutation. The mean age of mutation carriers was 52 years (SD 14) and for non-carriers 67 years (SD 13). Both mutation carriers and non-carriers were older than non-tested respondents with a mean age of 42 years (SD 17, *p* = 0.007). Twelve respondents from the 33 LS families were index patients.

**Table 1:** Characteristics of the respondents

	Mutation carriers	%	Non -carriers	*%*	Non tested respondents	%	Total respondents	%
Number of respondents	59		70		16		145	100
Male	26	44	24	34	6	38	56	39
Mean age (±SD)*	52 (14)		67 (13)		42 (17)		55 (15)	
*Marital status*								
Single	5	8	7	10	3	19	15	10
(As) married	46	78	53	76	9	56	108	74
Divorced/separated/widowed	7	12	8	11	4	25	19	13
Missing	2	3	1	1	0	0	3	2
*Number of children*								
None*	8	14	5	7	7	44	20	14
One or more children*	51	86	65	93	9	56	125	86
*Employed*								
Yes	38	64	30	43	10	63	78	54
Retired*	12	20	27	39	2	13	41	28
Student	1	2	1	1	1	6	3	2
Missing	2	3	2	3	0	0	4	3
*Education*								
High educational level	24	41	20	29	6	38	50	34
Low educational level	32	54	47	67	10	63	89	61
Missing	3	5	3	4	0	0	6	4
*Relation to index*								
Index patient	9	15	3	4	0	0	12	8
First degree relative*	24	41	27	39	3	19	54	37
Second degree relative*	25	42	33	47	10	63	68	47
Third degree relative	1	2	7	10	2	13	10	7
*Cancer diagnosis*								
Yes*	19	32	11	16	0	0	30	21
*Anxiety and cancer worry*								
Mean cancer worry (±SD)	5.3 (1.4)		5.2 (1.5)		5.1 (1.2)		5.1 (1.4)	
HADS anxiety (±SD)	4.1 (3.5)		4.7 (3.6)		4.0 (3.3)		4.5 (3.5)	
Median number of relatives with LS cancers*	2		2		1*		2	

* *p* = < 0.05, non-tested respondents vs LS mutation carriers and non-carriers

### LS mutation carriers and non-carriers compared with non-tested respondents

Demographic and family characteristics of mutation carriers, non-carriers and non-tested respondents are shown in Table 1. Non-tested respondents differed from LS mutation carriers and non-carriers in age, number of children, degree of relatedness to the index patient and cancer diagnosis. Of non-tested respondents 44 % did not have children, compared to 14 % of mutation carriers (*p* = 0.013) and 7 % of non-carriers (*p* = 0.02).

Twelve (8 %) respondents had been index patients within their family and thus the first informed on LS in the family. Fifty-four respondents (37 %) were first-degree relatives of the index patients and 78 (53 %) were second or third-degree relative of the index patient. More non-tested respondents (63 %) were second-degree relatives of the index patient, compared to mutation carriers (42 %, *p* = 0.03) and non- carriers (47 %, *p* = 0.02). A minority of total respondents (N = 10) were third degree relatives (Table 1). None of the non-tested respondents reported to be diagnosed with cancer, while 19 (32 %; *p* = 0.004) of the mutation carriers and 11 (16 %; *p* = 0.116) of the non-carriers reported to be diagnosed with cancer. Furthermore, non-tested family members reported to have a median of one relative with LS-associated cancer, while tested relatives had a median of two relatives with cancer (*p* = 0.01).

HADS scores did not differ between non-tested respondents and LS mutation carriers and non-carriers (mean HADS respectively 4.0; 4.1; 4.7, Table 1) and are comparable with the mean HADS scores of the Dutch general population between 18 and 65 years of age [39]. Fourteen respondents (10 %, six mutation carriers, seven non-carriers, one non tested respondent) had an anxiety score ≥11 and two other respondents (1 %, one non-carrier, 1 non-tested) had a depression score ≥11. Mean worry about cancer did not differ among mutation carriers, non-carriers and non-tested respondents (Table 1).

### Experiences with the family mediated approach

Table 2 shows the experiences with the family-mediated approach. A total of 115 of the 145 (79 %) respondents were informed by a family member about the diagnosis LS mostly by means of a personal explanation (70/145; 48 %) and/or the letter provided by the Clinical Genetics department to the index patient (63/145; 43 %). Interestingly, five of sixteen non-tested respondents reported to be informed on LS diagnosis by a genetic counsellor. In three cases it was confirmed in our institutional LS database that these cases were counselled but refrained from genetic testing.

**Table 2:** Experiences with the family-mediated approach

	Mutation carriers	%	Non carriers	%	Non- tested respondents	%	Total respondents	%
*2a. Communication within the family, answered by all respondents*								
Number of respondents	59	100	70	100	16	100	145	100
*When were you informed about LS in your family?*								
<1 week after diagnosing LS in a family member	20	34	18	26	3	19	41	28
<1 month after diagnosing LS in a family member	8	14	13	19	3	19	24	17
<6 months after diagnosing LS in a family member	3	5	11	16	1	6	15	10
<1 year after diagnosing LS in a family member	3	5	10	14	0	0	13	9
<5 years after diagnosing LS in a family member	6	10	3	4	3	19	12	8
>5 years after diagnosing LS in a family member	2	3	1	1	0	0	3	2
Missing	17	29	14	20	6	37	37	26
*Informed on the diagnosis of LS in the family by…(multiple answers)*								
Family member	41	–	61	–	13	–	115	–
Clinical geneticist/counsellor	31	–	17	–	5	–	53	–
Missing	7	–	0	–	0	–	7	–
*Communication tools within the family (multiple answers)*								
Family information letter genetics	24	–	34	–	5	–	63	–
Personal letter from a family member	6	–	12	–	0	–	18	–
Personal explanation from a family member	25	–	34	–	11	–	70	–
Missing	4	–	2	–	0	–	6	–
*2b.* *Experiences on being informed by a relative about LS*								
Questions are answered by family members who answered to be informed by a relative about LS								
Number of respondents	41	69	61	87	13	81	115	79
Which family member informed you about LS?**								
First degree family member	31	75	38	62	12	92	81	70
Second degree family member	6	15	6	10	1	8	13	11
Third degree relative	4	10	17	28	0	0	21	18
Missing	0	0	0	0	0	0	0	0
Contact with the informing family member**								
Poor	6	15	13	21	3	23	22	19
Neutral	5	12	12	20	0	0	17	15
Good	29	71	35	57	10	77	74	64
Missing	1	2	1	2	0	0	2	2
Effect on family relations**								
Family relations improved	4	10	3	5	1	8	8	7
Family relations worsened	2	5	0	0	1	8	3	3
No change in family relations	34	83	55	90	9	69	98	85
Missing	1	2	3	5	2	15	6	5
Burdensome being informed by family members**,^a^								
Burdensome*	14	34	4	7	1	8	19	17
Moderately burdensome	16	39	24	39	6	46	46	40
Not burdensome	11	27	30	49	6	46	47	40
Missing	0	0	3	5	0	0	3	3
*2c.* *Experiences on informing relatives about LS.*								
Questions are answered by respondents who answered to have informed a relative or relatives about LS								
Did you inform a family member about the diagnosis of LS in your family								
Yes	35	56	35	47	4	25	74	51
No	24	41	32	46	12	75	68	47
Missing	0	0	3	7	0	0	3	2
Number of respondents	35	59	35	50	4	25	74	51
Burdensome to inform family members***^,a^								
Burdensome	10	28	5	14	1	25	16	22
Moderately burdensome	16	46	11	32	1	25	28	38
Not burdensome	9	26	19	54	2	50	30	40
Missing	0	0	0	0	0	0	0	0

^a^ Converted to 3-point Likert scale

* *p* = < 0.05, LS mutation carriers vs non-carriers

** Answered by respondents who answered to be informed by a relative about LS

*** Answered by respondents who answered to have informed a relative about LS

The majority of the respondents, who were informed by a family member about the presence of LS in their family, were informed by a first degree family member (81/115; 70 %) and most of them (74/115; 64 %) reported to have good contact with this family member. For most respondents the LS disclosure did not change their contact with the family member. The majority of respondents informed by a family member about LS (65/115; 57 %) reported that they had experienced the process of being informed by a family member as (moderately) burdensome. Significant more mutation carriers than non-carriers reported burden due to being informed on the LS diagnosis by a family member (*p* = 0.002). Moreover, more mutation-carriers than non-tested respondents experienced burden while informing other family members about LS, but this difference was not significant (*p* = 0.07).

Seventy-four respondents (51 %) answered they had informed a relative about LS themselves. The majority (44/74; 59 %) of these had experienced this as (moderately) burdensome.

### Attitudes towards the family mediated approach

Most respondents (106/145; 73 %) reported to be satisfied with the current family-mediated approach of communicating LS diagnosis within the family (Table 3). Of the 30 respondents (21 %; 15 mutation carriers; 12 non- carriers; 3 non-tested) who did not agree with the current family mediated approach, 23 (77 %) respondents preferred being informed by a medical specialist. The 30 respondents, disagreeing with current family mediated approach belonged to sixteen LS families. In these sixteen families two till four family members per family shared the opinion that not family members but health professionals should inform relatives about LS diagnosis. Women more often than men reported that health professionals should inform relatives (28 vs 14 %).

**Table 3:** Attitudes towards the family-mediated approach

	Mutation carriers	%	Non - carriers	%	Non tested respondents	%	Total respondents	%
Do you think another way of informing relatives on Lynch syndrome is needed?								
No, current procedure is sufficient	41	69	54	77	11	69	106	73
Yes	15	25	12	17	3	19	30	21
I would have liked to receive no information about LS	1	2	1	1	1	6	3	2
Missing	2	3	3	4	1	6	6	4
Respondents who did not agree with the current procedure, suggested to be informed by:								
Medical specialist at the hospital	15	100	12	100	3	100	30	100
General practitioner	12	80	9	75	2	67	23	77
Family meeting	1	7	1	8	1	33	3	10
Opinion of all respondents towards statement I:	2	13	2	17	0	0	4	13
It is the personal duty of LS mutation carriers to inform one’s family members								
Disagree*	1	2	2	3	2	13	5	3
Neutral	7	12	7	10	5	31	19	13
Agree*	51	86	59	84	9	56	119	82
Missing	0	0	2	3	0	0	2	1
Opinion of all respondents towards statement II:								
It is the moral duty of physicians to inform patients in case of Lynch syndrome *in their family*								
Disagree	6	10	12	17	4	25	22	15
Neutral	14	24	9	13	1	6	24	17
Agree	35	59	46	66	11	69	92	63
Missing	4	7	3	4	0	0	7	5

* *p* = < 0.05, non-tested respondents vs LS mutation carriers and non-carriers

Furthermore, the majority of the respondents agreed with the statement that it is the moral duty of healthcare specialists to inform individuals about LS in their family (63 %). Also, most respondents agreed that it is the personal duty of LS family members to inform relatives about LS (82 %) However, significantly more of the non-tested respondents did not agree that it is the personal duty of tested individuals to inform the family about the LS diagnosis in their family compared to tested respondents (13 % of non-tested respondents vs 2 % of mutation carriers and 3 % of non- carriers, *p* = 0.004).

### Motivation for genetic testing for LS

**Fig. 2:**
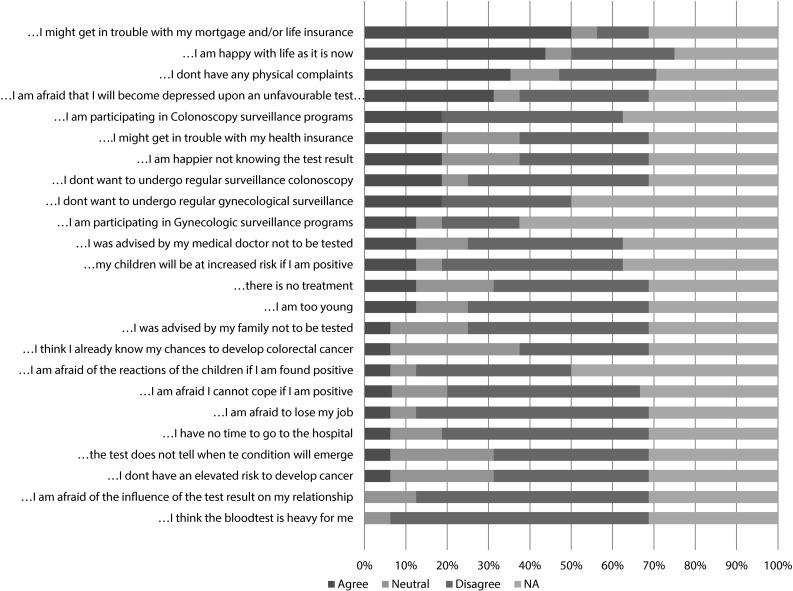
Motivations of non-tested respondents for not opting for the genetic test for LS (N = 16), NA = not available

The most important reasons for genetic testing were: (1) availability of surveillance programs for LS (61 %), (2) preference to end insecurity regarding LS diagnosis (34 %), and (3) fear for cancer (14 %, Table 4). The three most important reasons for declining genetic testing by non-tested respondents were: (1) worry that testing would lead to problems with life insurance and mortgage (50 %), (2) being content with life as it is (44 %), and (3) not experiencing any physical complaints (37 %, Fig. 2). Fear for surveillance programs was reported in 19 % of non-tested respondents.

**Table 4:** Motivations for uptake of genetic testing for LS (N = 129), >100

Motivation tested respondents	Mutation carriers	%	Non-carriers	%
Fear for cancer	8	14	10	14
Availability of surveillance programmes for LS	36	61	21	30
To end insecurity regarding LS diagnosis	20	34	31	44
Other	11	19	9	13

## Discussion

In this cross-sectional survey among 145 individuals from LS families, we evaluated the current family-mediated procedure for informing at risk relatives about the identified familial LS mutation. Although the majority of the respondents were satisfied with the current family-mediated approach of communicating LS diagnosis within the families, we found that a majority of the respondents (57 %) experienced being informed by a family member as (moderately) burdensome. Moreover, approximately half of the respondents experienced informing a family member about the LS diagnosis as (moderately) burdensome as well. Fortunately, for the vast majority of respondents being informed by a family member did not have an adverse impact on the relationship with that family member.

Twenty-one percent of the respondents would prefer another way of informing relatives on LS. Most of these respondents thought family members should be informed directly by a medical specialist. This percentage is in agreement with previous results of studies of Aktan-Collan et al. [29] and Pentz et al. [40], who reported that 25 and 29 %, respectively, would prefer another way of informing relatives by the family-mediated approach.

We found that half of the respondents who preferred another way of informing reported that more members in their family shared this opinion. It may be that family culture plays a role in person’s preferred method of informing relatives. Families in which communication is less open or in which family relationships are less intimate may experience informing relatives about LS as more burdening. In line with Aktan-Collan et al., we also observed gender differences in attitude towards informing relatives. We found that women more often than men reported that health professionals should inform relatives (28 vs 14 %). Previous studies have suggested that this fact is related to gender-related roles and communication patterns in the families. Women tend to be the most influential persons in the family network, and therefore may perceive more responsibility for communication of the diagnosis. Women may be more likely to perceive responsibility while society depicts them as natural ‘carers’ and may be more often the one that communicate with intimates about emotional topics in general [41, 42]. Another explanation for the finding of more often women informing family members may be the fact that endometrial cancer is the second malignancy in LS, which may be more difficult to discuss by males [29].

Informing all at-risk relatives about LS is of great importance, in order to enable each family member to make an informed decision about genetic testing, in particular because surveillance has proven to reduce morbidity and mortality from colorectal cancer [43]. Although all non-tested respondents in the current study were informed about LS in the family, it has been observed in a recent study that the LS diagnosis was less likely to be communicated to distant relatives [28]. Therefore, it is important to conduct further research on optimal methods to inform all at-risk family members, including more distant relatives. Decision aids are an innovative strategy for patient education and proposed to help optimally inform at-risk relatives and support them in their decision about genetic testing for LS. Currently, only one study has evaluated a paper-based decision aid for genetic testing for LS [44]. The results of this randomized trial were promising, since it has been found that the decision aid, in comparison with a control pamphlet, lead to lower decision conflict and increased informed decision making.

Furthermore, we evaluated moral and personal duties concerning informing relatives. In our study we found that the majority of the respondents agreed with the statement that it is the personal duty of LS family members to inform at risk relatives about LS in the family. On the other hand a smaller majority agreed that it is the moral duty of healthcare specialists to inform individuals about LS in their family. These findings bring up the complexities associated with current practice, in which the patient is primarily responsible to inform-at risk family relatives. For an extensive consideration of the medical, psychological, ethical and juridical aspects related to this topic, and the development of the current guidelines for clinical geneticists, we refer to the paper of Menko et al. [45]. The current guidelines underline the importance of the provision of written material and psychological support to help the patient informing family members and to overcome barriers in this potential difficult task.

Noteworthy, significantly more non-tested relatives as compared to tested relatives did not agree that it is the personal duty of tested individuals to inform the family about the LS diagnosis. It would be interesting to conduct an interview study among non-tested individuals to gain more insight in their attitudes towards the most optimal method of being informed about hereditary cancer.

In the current study, all non-tested respondents were informed about LS in the family and, consequently not being aware of LS diagnosis was not a reason for refraining from genetic testing. Reported reasons for refraining from genetic testing included problems with life insurance and mortgage, being happy with life as it is and not experiencing any physical complaints. The first two reasons are in agreement with previous studies on other hereditary cancer syndromes [38]. In the Netherlands, insurance companies are restricted in the use of genetic information of their clients by the Medical Examination Act., nevertheless, some people encounter problems when applying for insurance. Although this subject is included in the genetic counselling procedure, there is more need for clear information for the counselees on this topic. Furthermore, not experiencing any physical complaints was a common reason to refrain from testing in our study, which underlines the importance of counselling about LS in order to improve understanding on LS and available surveillance programmes.

Non-tested respondents differed from tested respondents on several demographic, medical and family characteristics. We found that non-tested respondents were younger and were less likely to have children than tested respondents. Consistent with this finding, it has been reported that knowledge about the risk for children is one of the main reasons for testing [46]. Furthermore, none of the non-tested respondents were diagnosed with cancer themselves, and, compared with tested respondents, had less family members with LS-associated cancers, and were less closely related to the index patient. These factors might influence how one experiences the threat of cancer and, subsequently, the urge to participate in genetic testing for LS. Genetic test decliners may benefit from information and counselling, even if they decide not to have a predictive genetic test. Fortunately, non-tested respondents were not found to be more vulnerable in terms of anxiety or cancer worries as compared to tested respondents.

Our study had a few limitations. First, the response rate among tested individuals was high (73 %), however the response rate among non-tested individuals was only 39 %. As in other studies, it is very difficult to include non-tested relatives [47]. Since relatives were asked to contact non-tested individuals, there may be a selection bias in that relatives with whom there was more intimate contact were more likely to be approached. Also, it is possible that non-tested relatives who cope with the worries about the risk of LS by avoiding the subject were less likely to participate in the current study. Nevertheless, this is the first study focussing on the specific group of non-tested relatives, which is known to be a very difficult group to approach. It provides new insight in the characteristics and motivations of non-tested relatives. Second, further qualitative research should be done in order to gain a deeper understanding of family interactions and communication and decision making about genetic testing for LS.

In conclusion, the current family-mediated procedure is accepted by the majority of LS family members, although a substantial proportion experienced burden informing relatives or being informed by relatives about LS. Healthcare workers should therefore carefully explore how index patients would experience communicating the LS diagnosis to family member, and whether a patient would prefer more involvement of the healthcare workers in informing relatives about LS, genetic testing and available surveillance programmes. Special attention should be paid to communication of LS to more distant relatives. It is important that family members who refrain from genetic testing are optimal and adequately informed about their own risks. They should be aware of the risks for LS, cancer and absence of symptoms in early stage cancer. Future studies should clarify risk perception of individuals who do not reach genetic services and the information and support needs of these individuals should be explored, including (online) decision aids.
